# Multi-label literature classification based on the Gene Ontology graph

**DOI:** 10.1186/1471-2105-9-525

**Published:** 2008-12-08

**Authors:** Bo Jin, Brian Muller, Chengxiang Zhai, Xinghua Lu

**Affiliations:** 1 Department of Biostatistics, Bioinformatics and Epidemiology, Medical University of South Carolina, 135 Cannon Street, Charleston, SC 29425, USA; 2 Department of Computer Science, University of Illinois at Urbana-Champaign, Urbana, IL 61801, USA

## Abstract

**Background:**

The Gene Ontology is a controlled vocabulary for representing knowledge related to genes and proteins in a computable form. The current effort of manually annotating proteins with the Gene Ontology is outpaced by the rate of accumulation of biomedical knowledge in literature, which urges the development of text mining approaches to facilitate the process by automatically extracting the Gene Ontology annotation from literature. The task is usually cast as a text classification problem, and contemporary methods are confronted with unbalanced training data and the difficulties associated with multi-label classification.

**Results:**

In this research, we investigated the methods of enhancing automatic multi-label classification of biomedical literature by utilizing the structure of the Gene Ontology graph. We have studied three graph-based multi-label classification algorithms, including a novel stochastic algorithm and two top-down hierarchical classification methods for multi-label literature classification. We systematically evaluated and compared these graph-based classification algorithms to a conventional flat multi-label algorithm. The results indicate that, through utilizing the information from the structure of the Gene Ontology graph, the graph-based multi-label classification methods can significantly improve predictions of the Gene Ontology terms implied by the analyzed text. Furthermore, the graph-based multi-label classifiers are capable of suggesting Gene Ontology annotations (to curators) that are closely related to the true annotations even if they fail to predict the true ones directly. A software package implementing the studied algorithms is available for the research community.

**Conclusion:**

Through utilizing the information from the structure of the Gene Ontology graph, the graph-based multi-label classification methods have better potential than the conventional flat multi-label classification approach to facilitate protein annotation based on the literature.

## Background

A thrust in bioinformatics is to acquire and transform contemporary knowledge from biomedical literature into computable forms, so that computers can be used to efficiently organize, retrieve and discover the knowledge. The Gene Ontology (GO) [1] is a controlled vocabulary used to represent molecular biology concepts, which is the *de facto *standard for annotating genes/proteins. The concepts in GO, referred to as GO terms, are organized in directed acyclic graphs (DAGs) to reflect hierarchical relationships among concepts. Currently, the process of extracting biological concepts from biomedical literature to annotate genes/proteins is manually performed by domain experts, whose roles are indispensable to ensure the accuracy of the acquired knowledge. However, the rate of manual annotation is outpaced by the growth of information in the biomedical literature [2]. Automatically performing literature-based GO annotation has drawn wide attention from the biomedical text mining community [3-8]. In addition to numerous publications by individual researchers, a special track was devoted to the task in the BioCreative conference in the form of a challenge from the biomedical text mining community [3]. Similar tasks were also investigated in the genomic track of the Text REtrieval Conference (TREC) [4].

Generally, the task of GO annotation based on free text of the literature can be cast as a text classification problem. Given a protein and the literature associated with it, one can potentially annotate the protein according to the classification (labeling) of the literature, for which various supervised classifiers can be trained, with the GO terms as target classes and the tokens in the training texts as input features. Due to the hierarchical nature of the GO concepts, GO annotation is also intrinsically a multi-label classification problem in that, when a protein is annotated with a GO term *t*, it is also considered to be annotated with all ancestors of *t*. A common approach to deal with multi-label classification in the machine learning field is to train multiple one-vs-rest binary classifiers, such that each classifier learns to discriminate cases of one class from the remaining classes [9]. Given a test case, all classifiers in such a system are invoked to make calls, and the case is labeled with the classes which turn out to be positive. Although such an approach can be adopted to perform GO annotation, it ignores the structure of GO and suffers from the following shortcomings. Firstly, the unbalanced training cases make learning difficult. This is because the number of training cases for an individual class is usually much smaller than the number of cases of all other classes combined in a multi-label classification scenario. Secondly, the outputs of such a system might not be compatible to the existing structure of classes, e.g., a case is labeled with a class, *c*, but not the parents of *c*.

Hierarchical classification takes into account the relationships among the target classes during training and outputs multi-labels that comply with the class relations. Hierarchical classification has received growing attention in the machine learning field in recent years [10-13]. In the bioinformatics domain, the hierarchical structure of GO was utilized to classify proteins based on various biological data, e.g., gene sequences and microarray [10,14,15]. With respect to literature-based GO annotation, reports from text mining workshops have explored hierarchical text classification for GO annotation, e.g., BioLink [16] and BioCreative [3,17]. In the study by Kiritchenko *et al *[16], a hierarchical classification system was built with AdaBoost algorithms as base classifiers. On the other hand, Verspoor *et al *[17] attempted to classify documents by utilizing the GO hierarchy structure to identify a set of candidate GO terms. In our study, we investigated and evaluated the performance of hierarchical classification systems built with state-of-the-art text classification methods, namely the support vector machine (SVM) and naïve Bayes classifier. In addition to conventional hierarchical classification, we also introduced a novel stochastic classification algorithm, referred to as random GO walk (RGOW), to perform probabilistic, graph-based multi-label classification. The motivation for RGOW is, by employing a stochastic mechanism, to alleviate the potential local maximum problem that results from the greedy search of top-down hierarchical classification.

The main goal of this study is to systematically investigate and evaluate the advantage, or lack of it, of a general class of graph-based multi-label classification methods (based on directed or undirected graphs). More specifically, we have studied the conventional non-hierarchical multi-label classification for GO annotation, the RGOW algorithm, and two top-down hierarchical classification algorithms. Our results show that graph-based multi-label classification methods significantly enhance the classification performance evaluated with metrics that measure exact matches. In addition, our methods are also capable of suggesting GO annotations closely related to the original annotations on the GO graph, even when they fail to predict them directly.

## Results

### PubMed augmented GO graph

In this study, the task of literature-based gene/protein annotation was cast as a graph-based classification problem. We constructed a PubMed augmented GO graph (see the Methods section) using the Biological Process branch of the GO combined with the Gene Ontology Annotation (GOA) [18] corpus. In this graph, a node represents a GO term, an edge represents the semantic relationship between a pair of GO term, and the structure of the graph follows the definition of the Biological Process ontology from the Gene Ontology Consortium. In addition, we further augmented the information of the graph by adding sets of PubMed identification numbers to each GO node as attributes of the object. This enables us to further associate each GO node with a text classifier to perform graph-based classification. Although we only studied the performance of graph-based classification on the Biological Process domain of the GO, the results would likely generalize to the Molecular Function and Cellular Component domains because the tasks are essentially the same.

**Figure 1:**
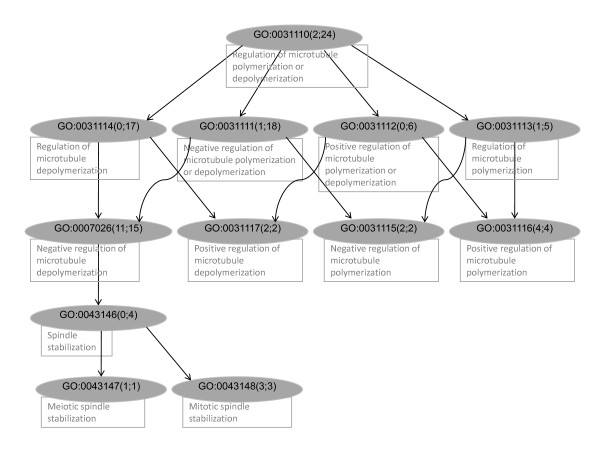
**A subgraph of the PubMed augmented GO graph constructed using the GOA data set**.

Figure 1 shows a subgraph of the PubMed augmented GO graph, illustrating hierarchical relationships between GO terms (nodes) organized as a DAG. Each node is associated with two sets of PMIDs: a set of PMIDs explicitly associated with the node, referred to as *nodeUniqPMIDs*; and a set consisting of all PMIDs associated with the node and its descendants, referred to as *nodeTotalPMIDs*. The cardinalities (sizes) of the *nodeUniqPMIDs *and *nodeTotalPMIDs *sets are shown (in Figure 1) as numbers within the parentheses next to the GO terms; the definitions of nodes are shown in the text boxes below the nodes.

**Figure 2:**
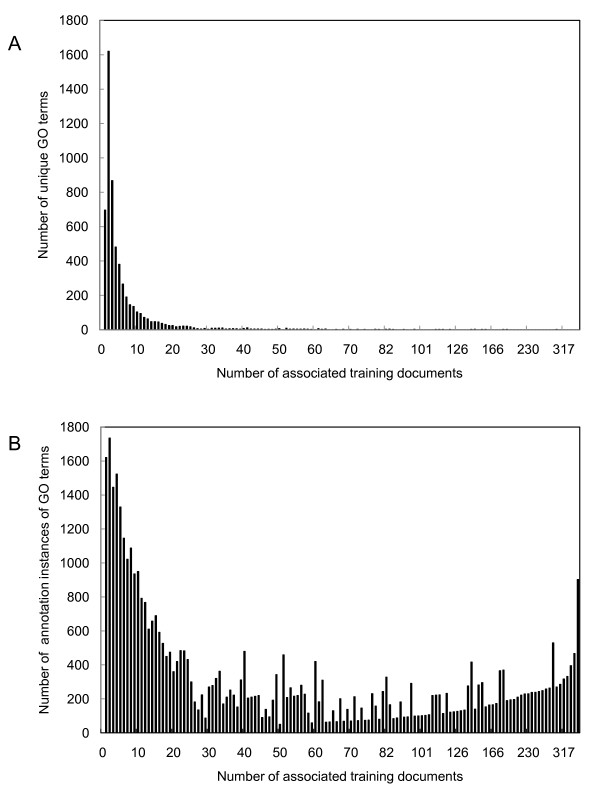
**Summaries of GO terms with respect to the number of training documents**. Panel A. The histogram of the unique GO terms grouped according to the number of training documents associated with each GO term. Panel B. The count of annotation instances of the GO terms grouped according to the number of training documents associated with them.

We further investigated the distribution of PubMed documents over the GO graph, which provides information on the state of current manual GO annotation processes, the degree of difficulty of training a literature-based GO annotation algorithm, and the motivation for graph-based classification. In Figure 2, Panel A shows the histogram of the unique GO terms grouped according to the number of training documents associated with each term (the cardinality of the unique GO terms' *nodeTotalPMID*s). It can be seen that many GO terms are associated with fewer than 10 training documents. One may reason that it is very difficult (if possible at all) to train accurate and generalizable text classifiers for the GO terms with so few training documents. Therefore, a more effective approach is to pool the training cases from these nodes to their ancestors and train more reliable classifiers at the ancestor nodes, which naturally leads to the graph-based multi-label classification approach. Panel B of Figure 2 shows the count of annotation instances of the GO terms, grouped according to the number of training documents associated with them. It can be seen that, although a relatively small number of GO terms have more than 20 training cases, the instances of observing these GO terms constitute a fairly large portion of all observed GO annotations. Thus, enhancing the capability of correctly predicting these GO terms will have a great impact on the overall performance of the classification systems.

### Performance evaluation

#### Evaluation of multi-label classification

Since the Gene Ontology Consortium adopts a principle of annotating proteins with GO terms that are as specific as possible, the observed GO terms in the GOA documents are usually the leaves of multi-label subgraphs. In order to evaluate multi-label classification, we reconstructed a multi-label subgraph for each test document based on its true/predicted GO annotations. The steps for constructing such a subgraph are as follows: 1) map a test document's GO annotations onto the PubMed augmented GO graph; 2) find the shortest path between the root and each of the true/predicted GO annotations; 3) join the paths using a union of the edges of the paths to make a subgraph of GO.

**Figure 3:**
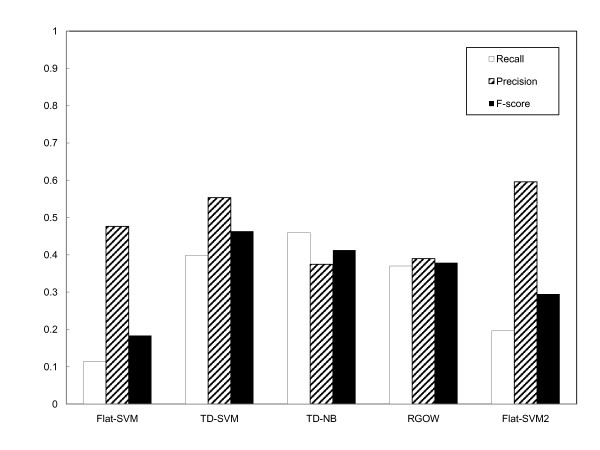
**The performance of flat-SVM, TD-SVM, TD-NB, RGOW and flat-SVM2 evaluated with multi-label classification evaluation (graph-to-graph) in terms of recall, precision and F-score**.

For graph-based multi-label algorithms, we used the outputs of each classification system as leaves to reconstruct the multi-label subgraph. For flat-SVM, we used two ways to evaluate its outputs: one is directly using the system outputs in multi-label evaluation; the other is treating its outputs as leaves (same as other systems) and building the multi-label subgraphs. Using the metrics specifically designed for graph-based multi-label classification described in the Methods Section, we evaluated the performance of different classification algorithms, and the results are shown in Figure 3. In Figure 3, the first four groups represent the performance of the flat-SVM evaluated with the direct outputs, the top-down SVM (TD-SVM), the top-down naive Bayes (TD-NB), and the random GO walk (RGOW). From these four groups, it can be seen that the TD-SVM, TD-NB, and RGOW systems significantly outperform the flat-SVM, with folds of increase in recall and F-score. The last group (Flat-SVM2) in the figure is the performance of the flat-SVM evaluated on the multi-label subgraphs built based on its outputs. This procedure is equivalent to evaluating the result from a flat SVM classifier as if it is from a hierarchical classifier, even though it does not utilize the GO graph during training. It is interesting to see that, although its performance is better than that of the flat-SVM, the flat-SVM2 is outperformed by the two top-down algorithms and the RGOW in terms of recall and F-score. These results indicate that the better performances by the graph-based classifiers indeed resulted from utilizing information from the GO graph structure during training the classifiers, rather than due to the differences in evaluation procedures.

#### Leaf-to-leaf evaluation

**Figure 4:**
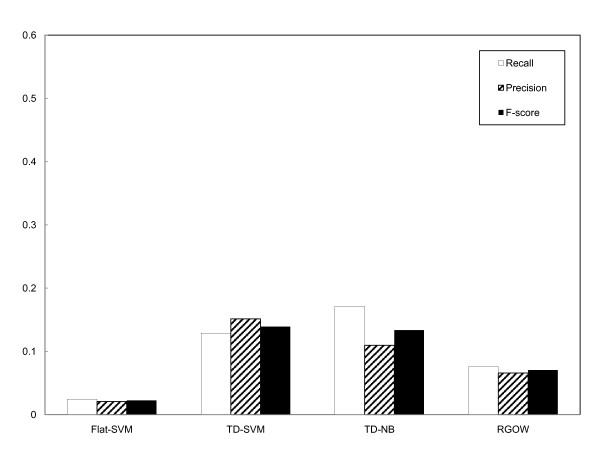
**Systems' performance evaluated with leaf-to-leaf evaluation in terms of recall, precision and F-score**.

The multi-label evaluation measures the accuracy of the systems by comparing subgraphs, such that it evaluates the overall capability of predicting both specific and general terms on the graph. In practice, protein annotation requires predicting the GO terms that are as specific as possible, and therefore we evaluated how accurately the predicted leaves (specific GO terms) matched the true annotations, a procedure referred to as leaf-to-leaf evaluation. The results are shown in Figure 4. Again, the results show that the graph-based multi-label classification methods significantly outperform the flat-SVM. TD-NB achieves a recall of around 17%; this recall represents that ~6,800 out of 40,000 instances of GO annotation in the GOA corpus were correctly predicted. It is interesting to note that precision for the flat-SVM decreases significantly in the leaf-to-leaf evaluation when compared to that in the graph-to-graph evaluation. This difference indicates that many of the correct predictions by the flat-SVM are general GO terms at the top levels of the GO graph, which can be detected in graph-to-graph evaluation. However, the flat-SVM is less capable of predicting more specific GO terms observed in the test cases, and thus it performs much worse in the leaf-to-leaf evaluation.

#### Evaluating performance using graph-based metrics

As shown in Figure 2, a large number of observed GO terms in the GOA corpus have only a few training documents, so it is almost impossible to train reliable classifiers for them. We conjectured that the misclassification of these cases (classes) constituted the majority of the test errors in multi-label classification. Instead of treating the misclassification of these terms as complete losses, it would be interesting to quantify and evaluate how closely the predicted and observed labels are located in the GO graph. One may argue that the loss incurred from predicting a label only one step away from the true label is more acceptable compared to predicting a label 5 steps apart from the true label. Indeed, one motivation of graph-based multi-label classification is to pool the training cases through training case propagation, so that it is possible to train more *reliable *classifiers associated with the ancestors of a GO node that has sparse training cases. Therefore we would like to evaluate how closely the predictions by these relatively reliable ancestor classifiers relate to the true classes. To this end, we devised graph-based metrics to evaluate results.

**Figure 5:**
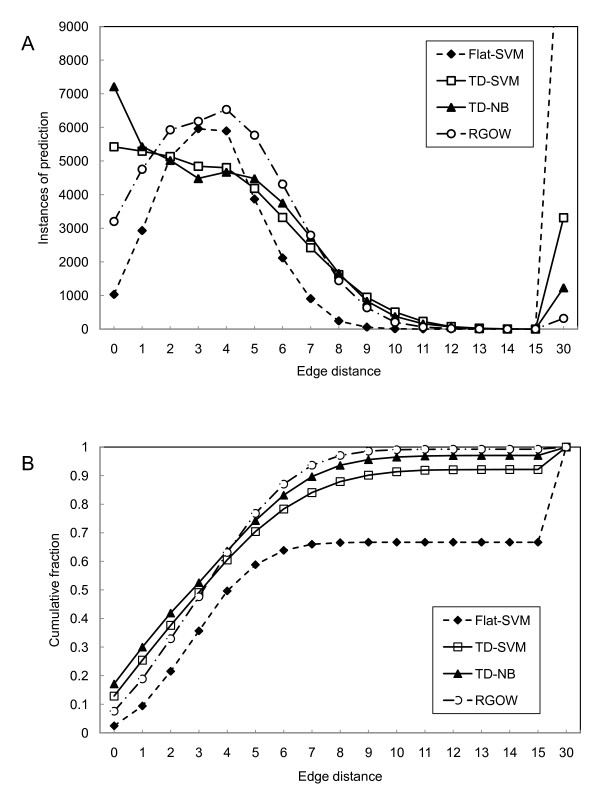
**Systems' performance evaluated with graph-based metrics**. Panel A. The distribution of the shortest distances of the predicted labels to the true GO annotations in the test set. Panel B. Cumulative percentile of GO terms with respect to the number of steps from the predicted labels. If a true class is missing from the predicted labels, the distance is set to 30.

**Figure 6:**
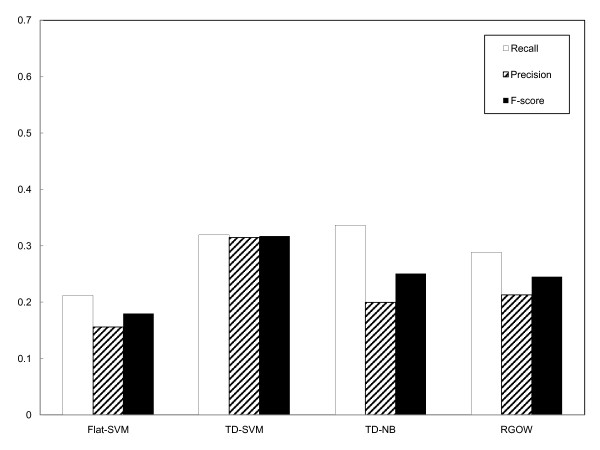
**Systems' performance in terms of recall, precision, and F-score for relaxed hits (within two steps)**.

During graph-based evaluation, for each true GO term in testing cases, we searched for the shortest path from the true label to the leaves of the predicted subgraph, and the number of edges in the path was used as a metric to reflect how close to the true label the predicted labels were. The shorter the path, the better the performance. Panel A of Figure 5 shows the distribution of the shortest distances of the predicted labels to the true GO annotations in the test set. Note that the paths with the length of zero reflect the correct predictions, and thus these numbers essentially agree with the recall of classification systems. It is interesting to note that many observed GO annotations are within one or two steps from the predicted multi-labels, and all graph-based classification systems perform better than the flat-SVM multi-label classification system. Panel B of Figure 5 plots the cumulative percentile of GO terms (y axis) with respect to the number of steps from the predicted labels. It can be seen that 33% – 42% of the true GO annotations are within only two steps from the labels predicted by the TD-NB, TD-SVM and RGOW. The results indicate that these graph-based classification systems are capable of predicting GO annotations very close to the true annotations, yet they are treated as misclassifications according to the conventional evaluation methods for multi-label classification. If we relax the criteria for correct predictions to include the predictions within two steps from the true labels, the graph-based systems can achieve even better performance (see Figure 6): 29% – 35% in recall, 20% – 31% in precision, and 24% – 32% in F-score. The results are encouraging given the difficulty of the classification problem for GO annotation.

#### Enhanced classification for classes with fewer training cases

**Figure 7:**
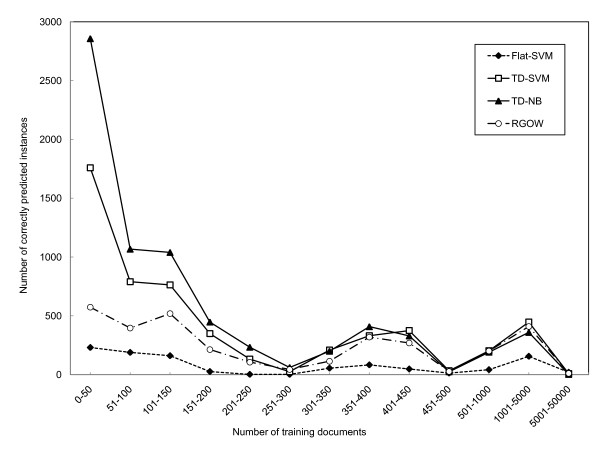
**The number of correctly predicted instances with training sets of different sizes**. For each method, the sum of these numbers is shown in Panel A of Figure 5 at edge distance equal to 0.

One of the motivations of employing graph-based classification methods is to address the problem associated with the training case imbalance that plagues flat classifiers. The assumption is that, by performing one-vs-rest classification locally rather than globally, the training case imbalance can be alleviated. To illustrate the impact of the size of training set on the prediction, we plotted the number of correctly predicted instances for each classification algorithm, grouped according to the number of training documents associated with each GO term in Figure 7. The figure illustrates that, for the GO classes with fewer than 50 training documents, the graph-based multi-label classification systems significantly outperform the flat multi-label classification method. As the number of training cases increases, the differences between the classification algorithms begin to diminish. These results indicate that the graph-based multi-label classification algorithms improve the performance on the classes with small training sets. These results are highly encouraging because GO terms with few training documents are the most difficult to predict.

## Discussion

In this study, we transformed the problem of literature-based prediction of GO annotation to a graph-based multi-label classification problem. Our results indicate that, through utilizing the structure of the GO graph, the graph-based multi-label classification algorithms significantly outperform the conventional flat multi-label classification approach. Furthermore, our results demonstrate that graph-based classification is capable of suggesting annotations that are semantically close to the true annotations. These results indicate that the graph-based multi-label classification methods have better potential than the conventional flat multi-label classification approach to facilitate protein annotation based on the literature.

Controlled vocabularies such as the GO and the Unified Medical Language System (UMLS) [19,20] provide computable forms of biomedical concepts, which are critically important in knowledge representation and are widely used in molecular biology and medicine. Interconnections between biological concepts can often be best represented as DAGs rather than trees. Although there have been many investigations on tree-based hierarchical text classification, studies of utilizing a graph structure for multi-label classification of text are few. Recently, Barutcuoglu *et al*. have proposed a sophisticated Bayesian network framework to perform graph-based hierarchical multi-label classification and employed it to predict GO annotations of proteins based on biological data, e.g., gene expression and protein-protein interactions [10]. Their framework requires a relatively large number of training cases in order to train their model, such that they limited the target classes to about 100 GO terms with at least 20 training cases. This requirement would have eliminated most biologically specific GO terms in our case. In contrast, our methods can be applied on the full graph of the Biological Process domain of GO.

Our work is closely related to that by Kiritchenko *et al *[16] in terms of problem formulation and evaluation. In their work, the investigators employed a global hierarchical classification system with an AdaBoost algorithm as the base classifier. In this study, we further investigated the performance of systems consisting of SVM and naïve Bayes classifiers, which are well established as the best text categorization classifiers [21]. In terms of evaluation, our graph-to-graph evaluation is essentially equivalent to the hierarchical recall and precision from Kiritchenko *et al*, in that they all evaluated the performance of overall multiple-label classification. In addition, we also performed the leaf-to-leaf evaluation which is more relevant to the real world evaluation from biologists' point of view. Furthermore, their evaluation concentrated on exact matches, which may not fully reflect the benefit of graph-based classification revealed by our relaxed graph-based evaluation. Thus, our evaluation methods demonstrated additional advantages of graph-based multiple-label classification to previous studies. Although it would be ideal to include their method in our evaluation, the lack of available software makes it difficult to perform a fair comparison due to potential minute variances in re-implementation.

Graph-based multi-label classification from this study is readily carried out as a series of localized classifications. For the TD-SVM and TD-NB algorithms, the localized classification is performed in a breadth-first-search manner, which is guaranteed to stop when all feasible paths are visited. In addition, to improve classification accuracy, employing top-down classification algorithms is more efficient due to their branch-and-bound nature. On the other hand, the RGOW algorithm transforms the DAG into an undirected graph and traverses the graph following the most probable paths. In addition to a more thorough search of the graph, the advantages of this algorithm also include the probabilistic outputs that accommodate the uncertainty of the predictions. Our results indicate that the probabilistic outputs by RGOW correctly reflect the uncertainty of predictions and can be further utilized to determine the decision threshold of classification.

The more important advantage of the graph-based multi-label classification algorithms lies in the fact that, even when not exactly matching the true target annotations, many of the predicted GO annotations are semantically close to the target annotations. This is the underpinning characteristic and motivation of our approach – suggesting and predicting annotations that are as close as possible to the GO terms with few training cases, and the classification on these GO terms would be impossible otherwise. Note that, since most of the observed GO annotations are very specific per the guidelines of the Gene Ontology Consortium, the predicted GO annotations that are only one step away from the true annotation should be fairly specific too. If these predictions are counted as correct, the systems can achieve around 0.4 in recall, which may potentially be helpful to human annotators during annotation processes.

Although outperforming the flat classification system, the current graph-based multiple-label classification methods need further improvement in order to meet the requirements of real-world literature-based annotation. Reasonable directions for improvement include, first, further fine-tuning the base classifiers. For example, one may fine tune and vary the classification threshold based on the level of the node. Second, a refined approach would use more specific training data. Ideally, the most relevant part of a document related to the GO terms should be identified through semantic analysis [22] and used for training classifiers.

## Conclusion

In this paper, we investigated and studied the methods of enhancing automatic multi-label classification of biomedical literature by utilizing the structure of the Gene Ontology graph. We systematically evaluated and compared three graph-based classification algorithms to a conventional flat multi-label algorithm and concluded that through utilizing the information from the structure of the Gene Ontology graph, the graph-based multi-label classification methods have better potential than the conventional flat multi-label classification approach to facilitate protein annotation based on the literature.

## Methods

### Data set

The Uniprot [23] gene-GO association file, version 47, was downloaded from the website of the Gene Ontology Annotation (GOA) [18] project of the European Bioinformatics Institute. Each entry in the association files contains a gene identification number, the associated GO term, and the PubMed identification number (PMID) for the annotation if available, and thus the data provide the link between the GO annotation and the literature. A corpus consisting of the titles and abstracts of 36,423 MEDLINE entries was downloaded from the National Center for Biotechnology Information (NCBI) using the Entrez E-utility service. The corpus was processed as follows: (1) common words from a standard English "stop words" list were removed; (2) words were stemmed using the Porter stemmer algorithm [24]; (3) words with fewer than 5 occurrences in the corpus were discarded, resulting in a vocabulary of 33,230 unique words. In this study, we only used the Biological Process branch of the GO to study the performance of the graph-based multi-label classification methods, and the approaches are readily extendable to other GO domains.

### Constructing the PubMed augmented GO graph

The GO definition file released in April 2007 was downloaded from the GO website and used to construct a GO graph. We have developed a Python software package referred to as GOGrapher (manuscript in preparation), which contains a set of application programming interfaces for building a GO graph and performing various graph-based queries. In the GO graph, each node (vertex) represents a GO term, and each directed edge corresponds to the IS_A relationship between a parent-child GO term pair. In the GOA corpus, each node is associated with a set of PMIDs, referred to as *nodeUniqPMIDs*. The GO graph was topologically sorted [25], and the PMIDs associated with each GO node were propagated from all children to their parents in a bottom-up fashion. At this stage, each GO node was associated with an additional set of PMIDs referred to as *nodeTotalPMIDs*, consisting of the union of its own *nodeUniqPMIDs *and its children's *nodeTotalPMIDs *sets. After propagation of PMIDs, the nodes with an empty set of *nodeTotalPMIDs *were pruned from the graph, which resulted in a graph with a total of 5,797 nodes (target classes). Based on the *nodeTotalPMIDs*, a word-vector was constructed for each GO node, of which each element was the count of the word associated with the GO term in the corpus. We refer to this graph as the PubMed augmented GO graph. A sub graph of the PubMed augmented GO graph is shown in Figure 1.

### Classification methods

#### Flat multi-label classification system

As a baseline reference classification system that would not utilize the structure of GO, a flat one-vs-rest multi-label classification system was constructed. SVM was chosen as the base binary classifier because it is the state-of-the-art classifier for text categorization [26-28]. In this model, the GO structure was flattened after propagation of PMIDs, and each class (node) was associated with a binary SVM classifier [26-28] to discriminate this class from the other classes. We refer to such a classification system as flat-SVM. A Python wrapper for LibSVM [29] with a linear kernel and default parameter settings were employed. Given a GO node, *g*, all PubMed documents in its *nodeTotalPMIDs*_*g *_set were labeled as positive training data and all other documents not covered by *nodeTotalPMIDs*_*g *_were labeled as negative training data.

#### Top-down hierarchical classification system

We designed and compared two classification systems for GO annotation with either SVM or naive Bayes as a base classifier. The classification procedure of the system is similar to top-down, tree-based hierarchical classification [12,30] but is generalized to deal with the more complicated GO graph structure. The idea underlying the top-down system was to perform localized one-vs-rest, rather than overall one-vs-rest classification at each level to overcome the training case imbalance problem. Given a GO node, *g*, a base classifier was trained with the documents of *nodeTotalPMIDs*_*g *_as positive training cases and the documents of *negTrainingSet*_*g *_defined in Equation (1) as negative training cases. Here, *negTrainingSet*_*g *_is the set of the union of all *g*'s parents total PMIDs excluding *g's nodeTotalPMIDs*.

(1) $$negTrainingSet_{g}=\underset{j\in parents(g)}{\cup }nodeTotalPMIDs_{j}-nodeTotalPMIDs_{g}$$

Naive Bayes is a well-studied probabilistic algorithm with robust performance on text classification. In this study, a multinomial version of naive Bayes [31] was implemented. Let *V *be the set of vocabulary of the corpus and *W*_*d *_be a sequence of words in document *d*. For the binary naive Bayes classifier of node *g*, the prior probability, *p*(*c*_*g*_), the conditional probability of observing a word, *p*(*w*|*c*_*g*_), and the posterior probability for a class are defined as follows:

(2) $$p(c_{g}=1)=\frac{\left|nodeTotalPMIDs_{g}\right|+1}{\left|\underset{j\in parents(g)}{\cup }nodeTotalPMIDs_{j}\right|+2},$$

(3) $$p(w|c_{g})=\frac{count{\left(w\right)}_{c_{g}}+\beta }{\sum_{w^{\prime }\in V}count{\left(w^{\prime }\right)}_{c_{g}}+|V|\beta },$$

(4) $$p(c_{g}=1|d)\frac{p(c_{g}=1)\prod_{w\in W_{d}}p(w|c_{g}=1)}{p(c_{g}=1)\prod_{w\in W_{d}}p(w|c_{g}=1)+p(c_{g}=0)\prod_{w\in W_{d}}p(w|c_{g}=0)}.$$

In Equation (3), $count{\left(w\right)}_{c_{g}}$ is the count of *w *in the training documents for a given class *c*_*g*_; *β *is the Laplace smoothing parameter [31], which was set to 0.001 in this study. With individual base-classifiers trained at each GO node, classification of a new document was performed according to Algorithm 1 in a top-down, breadth-first-search manner as shown in Table 1.

**Table 1:** Algorithm 1 Top-down classification algorithm

1	**inputs**
	*d*, a new document
	*G*, a GO graph with trained base-classifiers
2	**initialize**:
3	*PA *← {*root*} //Predicted GO annotation set
4	*Q *← {*root*} //Queue for breadth first search
5	**while ***Q *not empty
6	*n *← *Q*.pop()
7	*S *← *G*.children(*n*)
8	**for **each *c *in *S*:
9	*y *← *c*.predict (*d*)
	//Classify *d *using the base-classifier of *c*
10	if y == 1:
	//if prediction is positive
11	*Q *← union(*Q*, *c*)
12	*PA *← union(*PA*, *c*)
13	**end**
14	**end**
15	**outputs: ***PA*

#### Random GO walk (RGOW)

RGOW performs a stochastic search of the best multiple-labels for a given document, based on the Metropolis-Hastings algorithm [32] with a simulated annealing procedure. We designed RGOW to explore if stochastic procedures can be used to alleviate the local maximum problem due to the greedy search nature of the top-down SVM and naïve Bayes classifiers. In addition, the system also outputs a probability distribution over the leaf labels reflecting the posterior probability of the multiple-labels.

An intuitive explanation for the algorithm is as follows: imagine that an undirected version of the PubMed augmented GO graph constitutes a landscape, and a new test document *d *is allowed to stochastically traverse the landscape to search for the most probable labels for it. At each step, the document stays at current node *g *and looks for the next node g*. A candidate node g* is stochastically selected according to a proposal distribution *q*(g* | *g, d*) defined as Equation (5) and accepted according to Algorithm 2 in Table 2. Furthermore, a simulated annealing procedure enables the algorithm to search for the global maximum of the landscape–the most probable labels for the document. If an affinity function is chosen such that it reflects the likelihood of the GO term being used to annotate the document *d*, a probability distribution over the multi-labels of the graph can be obtained by counting the samples that stop at each GO node followed by a normalization procedure. A posterior multinomial distribution guiding the next step from *g *(line 9 and 10 in Table 2) is constructed locally through a Bayesian approach, in which the probability of the document reaching node g* in the next step is defined as Equation (5). The term *p(g|d) *in Algorithm 2 (at line 12 in Table 2) is defined as Equation (6).

**Table 2:** Algorithm 2 Random GO walk

1	**inputs:**
	*d*, a new document
	*G*, a GO graph with training cases
	*nSample*, the sample size
	*nMaxSteps*, the number of maximum steps
2	**initialize**:
3	*finalLeaves *← {}
4	*finalLeavesProbs *← {}
5	**for ***n *in 1: *nSamples*
6	*g *← initialize()
7	//Select initial GO node randomly
	**for ***s *in 1: *nMaxSteps*:
8	*T *← TempFunc(s)
9	*nbrs *← *G*.neighbors(*g*)
10	g* ← *q*(g*|*g, d*)
	//Sample from proposal distribution, g*∈{*g*, *nbrs*}
11	*u *← uniform [0, 1]
12	**if ***u *<*A *← min⁡(1,p(g∗|d)1Tq(g|g∗,d)p(g|d)1Tq(g∗|g,d))
13	*g *← g*
14	**end**
15	*finalLeaves *← union(*finalLeaves, curNode*)
16	**end**
17	*finaLeavesProbs *← calProbFromSample(*finalLeaves*)
18	**outputs: ***finalLeaves, finaLeavesProbs*

(5) $$q(g^{*}|g,d)=\frac{p(d|g^{*})p(g^{*}|g)}{\sum_{j\in neighbors(g)}p(d|j)p(j|g)},$$

(6) $$p(g|d)=\frac{p(d|g)p(g)}{\sum_{j\in neighbors(g)}p(d|j)p(j)+p(d|g)p(g)},$$

where the probability quantities in Equation (5) are defined as follows:

(7) $$p(g^{*}|g)=\frac{\left|nodeUniqPMIDs_{g}\right|+1}{\sum_{j\in neighbors(g)}\left|nodeUniqPMIDs_{j}\right|+1},$$

(8) $$p(g)=\frac{\left|nodeUniqPMIDs_{g}\right|+1}{\left|nodeTotalPMIDs_{root}\right|},$$

(9) $$p(w|g)=\frac{count{\left(w\right)}_{g}+\beta }{\sum_{w^{\prime }\in V}count{\left(w^{\prime }\right)}_{g}+|V|\beta },$$

(10) $$p(d|g)=\prod_{w\in W_{d}}p(w|g).$$

In the above equations, *V*, *W*_*d *_and *β *are the same as defined in binary naive Bayes, *count(w)*_*g *_is the number of words taking the value of *w *in the document set of *nodeTotalPMIDs*_*g*_, and *neighbors*(*g*) is the set of neighbor nodes of *g*. With the local proposal distribution determined, a new document can traverse the GO graph through sampling (random walk) to search for the most likely GO annotation (see Algorithm 2 in Table 2)

In Algorithm 2, the function *calProbFromSample *calculates the probability that document *d *stops at node *g *by dividing the number of samples whose final visited node is *g *by the total number of samples. We set the sample size to 40 and the number of steps of the random walk to 30. The simulated temperature is defined as Equation (11):

(11) *TempFunc*(*i*) = (*C *ln(*i *+ *T*_0_))^-1^,

where *T*_*0 *_is the initial temperature and *C *is a constant. *T*_*0 *_and C were set to 1.1 and 4, respectively.

### Evaluation

#### Semantic distance

In this study, we adopted a commonly used method to measure the semantic distance between a pair of GO terms, in which the difference between the information contents (IC) [33-36] of the GO terms was employed as a measure of the semantic distance. Here, the IC of a GO term *t *was calculated as: IC(*t*) = -ln *P*(*t*), where *P*(*t*) was the probability of observing the term, calculated as the number of annotation instances by the term divided by the total number of annotation instances. Then the semantic distance between a parent-child pair of GO terms, *t*_*p *_and *t*_*c*_, was determined as follows,

(12) *dist*(*t*_*p*_, *t*_*c*_) = |*IC*(*t*_*p*_) - *IC*(*t*_*c*_)|.

Note, that the IC-based semantic distance is not a metric distance in that it does not satisfy the triangle inequality, which potentially introduces errors during a search for the shortest path between a pair of GO terms. However, the operations of searching for the shortest paths between GO terms were performed in a consistent manner during the evaluation of all classification algorithms, and therefore we believe this characteristic of the IC-based semantic distance had no significant impact on the comparison of the results.

#### Multi-label evaluation metrics

Since abundant training and testing data are available, we employed a four-fold cross validation procedure in evaluation. Evaluation of multi-label classification is different from that of conventional binary classification. In this study, we adopted the information retrieval metrics that were modified for evaluating multi-label classification [9,16,37]. Let *D *denote the test corpus and *Y*_*d *_and *Z*_*d *_be the true and predicted label sets, respectively, for document *d*. The precision, recall and F-score for a classification system are determined as follows,

(13) $$precision=\frac{\sum_{d=1}^{\left|D\right|}\left|Y_{d}\cap Z_{d}\right|}{\sum_{d=1}^{\left|D\right|}\left|Z_{d}\right|},$$

(14) $$recall=\frac{\sum_{d=1}^{\left|D\right|}\left|Y_{d}\cap Z_{d}\right|}{\sum_{d=1}^{\left|D\right|}\left|Y_{d}\right|},$$

(15) $$F-score=\frac{2\times precision\times recall}{precision+recall}.$$

#### Graph-based evaluation metrics

When the predicted labels do not match exactly with the true labels, the above metrics consider such an error as a complete loss. However, in the graph-based classification scenario, we wanted to know whether the predicted classes were closely related to the true classes even if they were not direct matches. We used the length (in number of edges) of the shortest path (measured with IC) between true and predicted labels as a metric for evaluating the closeness of the predicted and true labels. The shortest paths between all pairs of true and predicted labels were found using Dijkstra's algorithm [38].

### Software

A Python package is available at:



## Authors' contributions

XL conceived the project, BJ carried out experiments, BM contributed to coding of the library. All authors contributed to experiment designs and manuscript writing.
